# Anxiety and Sleep Quality Amelioration in College Students: A Comparative Study between Team Sports and Individual Sports

**DOI:** 10.3390/bs12050149

**Published:** 2022-05-17

**Authors:** Chaoxin Ji, Jun Yang, Lin Lin, Song Chen

**Affiliations:** 1 Physical Education Department, Northeastern University, Shenyang 110819, China; 2 College of Information Science and Engineering, Northeastern University, Shenyang 110819, China; yangjun@mail.neu.edu.cn; 3 School of Social and Political Science, University of Glasgow, Glasgow G12 8QQ, UK; 2514029L@student.gla.ac.uk

**Keywords:** anxiety, sleep quality, team sports, individual sports, college students

## Abstract

Background: Anxiety is one of the most common mental disorders and is often accompanied by sleep disturbances. Studies have focused on the ameliorative effect of sports games on anxiety and its subsidiary issues. However, the effect on the improvement of such mental and physical disorders between individual sports and team group sports is not yet clear, especially regarding their effects on anxiety and sleep quality improvement. Therefore, this paper explores the improvement effects of individual sports and team sports participation on anxiety symptoms and sleep quality amelioration. Objective: To explore the effects of individual and team group sports participation on ameliorating college students’ anxiety symptoms and sleep quality. Methods: A total of 197 college students were sampled in the study. The self-rating anxiety scale (SAS) and Pittsburgh sleep quality index (PSQI) were used to assess the severity of anxiety symptoms and level of sleep quality. Participants were randomly divided into three groups: team sports, individual sports, and control groups. The distribution ratio of these groups was 1:1:1. Results: Generally, compared with the control group, the subjects in the team sports group and individual sports group had greater improvements in anxiety amelioration and sleep quality improvements. Specifically, the improvement effect between individual and team sports groups was different. To assess the resulting differences, improvements in anxiety symptoms and sleep quality were compared between the team sports group and the individual sports group with reference to the mean change in the control group. In the corrected model, the odds advantage ratio (OR) of anxiety symptom improvement after individual sports was 3.18 (CI 2.87–11.21), and the advantage OR of anxiety symptom improvement after team sports was 4.99 (CI 4.06–14.87). The advantage OR of sleep quality improvement after individual sports was 7.32 (CI 5.53–18.22), and the advantage OR of anxiety symptom improvement after team sports was 7.98 (CI 6.69–19.98). Conclusion: After 6 weeks of intervention, it was found that both team sports and individual sports improved anxiety symptoms and sleep quality with different effects. Team sports were better at improving anxiety, while individual sports and team sports shared the same improvement effect with no significant difference in sleep quality improvement.

## 1. Introduction

Anxiety incubates when people feel an impending threat [1]. It is a common psychiatric disorder, with a higher incidence in adults [2]. Many other physiological disorders are associated with anxiety symptoms, such as Parkinson’s [3] and heart disease [4]. In general, adults who suffer from anxiety symptoms are more prone to have medical conditions [5]. Anxiety is closely associated with sleep quality, and the latter is often taken as a critical parameter for the observation of the former’s improvement [6,7]. For instance, people with anxiety symptoms often experience accompanying depressive symptoms and sleep disorders [8,9]. Furthermore, it has been pointed out that anxiety could cause sleep deprivation leading to anxiety exacerbation [10]. Ng’s research shows that anxiety can affect the sleep quality of college students, and poor sleep quality affects college students’ academic performance [11]. Therefore, researchers are currently attaching great importance to the treatment of anxiety. Many anxiety treatment methods have been applied, with different pros and cons. Pharmacological treatment [12], cognitive-behavioral therapy (CBT) [13], and so on, are much applied, while some anxiety patients resist drug therapy because of its side effects. CBT, in turn, may keep people with anxiety disorders away from treatment because of the long waiting time.

On the other hand, physical exercise has received increasing attention as an inexpensive and non-traumatic intervention for anxiety disorders. The application of physical exercise to intervene in patients with anxiety symptoms has achieved a positive effect. For example, Philippot conducted a 6-week physical exercise intervention for teenagers and found that, after the physical exercise intervention, the anxiety symptoms of teenagers were effectively relieved, which proved the effectiveness of physical exercise in relieving anxiety disorders [14]. Ji intervened with college students by applying physical exercise of different intensities and frequencies, and found that the effects of physical exercise of different intensities and frequencies on anxiety symptoms were inconsistent [15]. Physical exercise also plays a role in improving sleep quality. For example, Plekhanova studied the relationship between physical exercise and sleep in the elderly. The study found that the physical exercise of the elderly has a significant relationship with sleep time, and more physical exercise can effectively promote the prolongation of sleep time in the elderly [16]. Saidi’s study found that 12 weeks of physical exercise intervention can have a beneficial effect on the sleep quality of individuals, and the effect can last for a long time [17]. At present, most studies focus on how physical exercise affects patients with anxiety symptoms, and many intervention methods are used, including team sports, strength training, and resistance training [18,19]. However, physical exercise interventions for individuals with anxiety symptoms have been mixed and even contradictory. For example, a metanalysis by Axelsdottir and Bellon showed that physical exercise has a limited effect on individuals’ anxiety [20,21], while Yin pointed out through meta-analysis that physical exercise can effectively improve individuals’ anxiety symptoms [22]. The reason for the different results is that the intensity of exercise caused by the different means of intervention was different across the studies. Another possible reason is that there are many types of sports, which can be divided into team sports and individual sports. Team sports are sports that require multiple individuals to participate and require constant communication to complete. By adopting individual sports to intervene patients with anxiety, this approach may neglect the critial importance and the effect of group cooperation. However, Yang’s study found that group rehabilitation training can more effectively relieve the anxiety symptoms of college students [23]. Strickland’s study found that co-contemplation behaviors in people with social disorder anxiety can also effectively relieve anxiety symptoms [24]. Physical exercise has a certain influence on sleep quality, which has been recognized by many studies [25,26]. For instance, researchers have compared the effects of team sports with the effects of individual sports on anxiety amelioration, but their research has focused on athletes, mainly comparing athletes’ anxiety status in training and stress from the competitive process. Their research shows that team athletes have fewer anxiety symptoms than individual athletes [27,28]. Therefore, there is currently a lack of high-quality studies comparing the effects of team sports and individual sports on improving anxiety in the general population. To effectively explore the effect of relieving the anxiety symptoms of college students, this study comparatively applied team sports and individual sports to students with anxiety symptoms to observe the effect on college students’ anxiety symptoms. The main purpose of this study was to compare the effect of team sports and individual sports on the relief of anxiety symptoms in college students. Additionally, to compare the effects of team sports and individual sports on the sleep quality of college students, we also analyzed the sleep quality of college students.

Our study assumed that both team sports and individual sports could effectively relieve the anxiety symptoms and improve the sleep quality of college students. Still, the effects were different, and the effect of team sports on improving sleep quality and relieving anxiety was better than individual sports.

## 2. Materials and Methods

### 2.1. Participants

When recruiting the number of subjects, we first counted the minimum sample size required for this study. Query Advisor 7.0 was used to count the required sample size. After Query Advisor 7.0 analysis, we found that at least 60 individuals should be counted as the minimum sample size for each group to achieve statistical qualification. To obtain enough samples, we expected an attrition rate of around 25% per group, so a minimum sample size of 225 was required.

**Figure 1:**
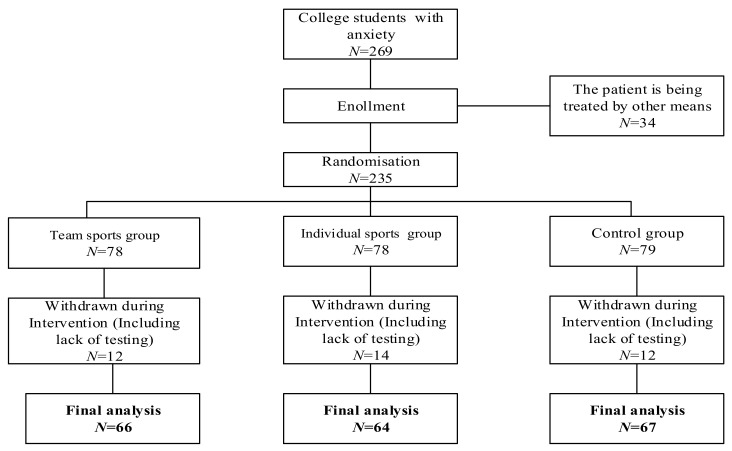
Participant flowchart across the study.

We recruited eligible college students from a mental health education center at a university in Northeast China. The diagnosis of anxiety disorders and other psychiatric disorders was performed by a psychiatrist prior to the sampling process in the mental health center to sample eligible college students. The inclusion criteria were as follows: (1) College students with anxiety disorders were screened through the Mini-International Neuropsychiatric Interview (Chinese version) (MINI). (2) The college students tested had no other physical diseases and were able to perform physical exercise. (3) After the physical exercise intervention, college students who regularly participated in physical exercise without our monitoring were excluded (participating in physical exercise two or more times per week). (4) College students who took pharmacological treatment were excluded. The flow of the experiment is shown in Figure 1. A total of 269 individuals agreed to participate and conduct a baseline assessment. After the screening process, 197 college students were randomly assigned to three groups. The average age of the college students was 21.3 years old, including 115 males and 82 females. The Body Mass Index (BMI) of the college students showed that most of them were overweight, and about 13% of the college students reported their smoking behavior. Most college students had had anxiety for less than 1 year. This study was approved by the Ethics Committee of Northeastern University (EC2020B019). The study procedure was in accordance with the ethical standards of the institutional and national research committee and with the 1964 Helsinki declaration and its later amendments or comparable ethical standards.

### 2.2. Study Methodology

Based on the research design, 197 participants sampled from the former criteria were randomly assigned to the three groups. According to the needs of the research, the first group of college students was defined as the team sports group, in which the intervention was carried out in the form of team sports. The second group was the individual sports group, which applied individual sports to intervene. The third group was the control group with no physical exercise intervention. All three groups separately set up three WeChat groups (a social software commonly used in China which can register attendance) and, in three groups, they all received twice-weekly counseling sessions on how to reduce anxiety. The content of the psychological counseling service was limited to the popularization of anxiety relief work, and the person in charge was the psychiatrist of the mental health education center. The psychiatrist’s intervention method was in the form of lectures on relieving anxiety symptoms in the WeChat groups. The three groups of college students all implemented the WeChat group sign-in function. If three or more sign-ins were missed due to personal and external reasons, the participant was not included in the final valid statistics.

The physical exercise groups had interventions twice a week for a total of 6 weeks of intervention. The intervention method used in the individual sports group was a combination of running exercises and strength training. The running was intervened by the Sports World Campus APP (campus running software), and the distance of each running was 2 km. Strength training included planks, sit-ups, push-ups, and elastic band exercises. After the college students finished running alone, they returned to the designated location for strength training. The overall duration was about 45 min, including 5 min of relaxation time. In the individual sports group, a Borg-rated perceived exertion (RPE) was used to monitor the exercise intensity of the college students. The exercise intensity was defined as moderate/high intensity when the RPE score was greater than 13 points. 

The team sports group exercised in the form of basketball games. Each time, they completed a 10 min warm-up before the game, and then played a 40 min basketball game. The team sports group was divided into multiple groups to compete against each other. The game adopted the standard basketball game rules. After the game was completed, the remaining 10 min comprised relaxation time and discussion time for the group. The topics of discussion were the degree of cooperation in this game, how to win in the next game, and summarizing the lessons of failure. Each participant was asked to sum up their experience and share them with the WeChat group. The college students in the third group did not engage in physical exercise intervention, but they posted about their anxiety and psychological status in the WeChat group twice a week.

During the monitoring process, if any participant from the two groups exercised privately outside of the monitoring procedure more than twice a week, at 30 min per time, this participant was excluded from the final sampling process (Figure 1). The first measurement was conducted before the formal intervention, and the last measurement was conducted after the end of the final intervention. If any participant from the control group participated in physical activity two or more times per week, for more than 30 min per exercise session, they were also excluded from the final measurement.

### 2.3. Test Main Results

Self-Rating Anxiety Scale (SAS): The anxiety level of the college students was monitored by the SAS. The SAS has a total of 20 items, each with a score of 1–4, with a total score of 20 to 80. The scale has been widely used [29]. In this test, the total score of SAS needs to be multiplied by 1.25 to fit China’s standard. Relevant studies have shown that SAS has shown good validity and reliability in Chinese anxiety measurement [30,31]. The SAS scores correspond to anxiety symptoms as follows: a score below 50 indicates normal; 50–59 indicates mild anxiety; 60–69 indicates moderate anxiety, and a score above 69 indicates severe anxiety.

Pittsburgh Sleep Quality Index (PSQI): The scale consists of 19 self-assessed items and 5 other-assessed items, of which the 19th self-assessed item and 5 other-assessed items are not scored. There were 7 components in the 18 items, which were sleep quality, sleep onset time, sleep time, sleep efficiency, sleep disorder, hypnotic medication, and daytime dysfunction [32]. The total PSQI score ranges from 0 to 21, with higher scores indicating worse sleep quality. A score between 0 and 5 indicates good sleep quality; a score between 6 and 10 indicates better sleep quality; a score between 11 and 15 indicates worse sleep quality; and a score between 16 and 21 indicates that sleep quality is poor. PSQI has a good application effect on sleep quality monitoring of Chinese residents [33,34].

### 2.4. Statistical Analysis

All statistical analysis was carried out using SPSS 26.0. Data are expressed as the mean, standard deviation, and 95% confidence interval (CI) of normal distribution, and the baseline and post-intervention changes are expressed as the average of the corresponding CI. The Chi-square test was used on descriptive data and the Kruskal–Wallis test on the baseline. For correlation analysis, we used the non-parametric Spearmann’s rank correlation and the parametric Pearson’s correlation, as indicated by *p*-values. The primary outcomes observed were the SAS and PSQI scores of college students. To evaluate the overall effectiveness and the effectiveness of the intervention group and the control group, an analysis of covariance (ANOVA) was used in the general linear model to produce a standardized average difference. To be able to assess whether the physical exercise intervention was effective, the mean change in SAS and PSQI pre- and post-test in the control group was used as a reference. According to the difference between the pre- and post-test scores in the control group, we defined the inter-group difference of SAS as 6 points (relief) and the inter-group difference of PSQI as 2 points (improvement). 

We determined the improvement of anxiety according to the average change score of the control group before and after the test. The three groups of college students were guided by psychologists; hence, it was possible to improve all three groups’ anxiety and sleep quality even though the control group did not undergo physical exercise. The ultimate purpose of this paper was to compare the effects of group exercise and individual exercise effects on anxiety and sleep quality amelioration. Therefore, psychological counseling factors in the individual and group team needed to be excluded. The excluding procedure was as follows: taking anxiety as an example, we subtracted the average anxiety score of all college students in the control group in the post-test from the average anxiety score of all college students in the control group in the pre-test, and finally obtained a difference of 6 points. If the score of any group of college students in the post-test was greater than 6 points compared with its pre-test, it was counted as anxiety amelioration. This method was also adopted in evaluating the improvement of college students’ sleep quality. SAS and PSQI binary logistic regression analyses were performed with multivariable adjustments in various models of confounding, and these variables, such as age, gender, BMI, and so forth (Table 1), were obtained at the baseline measurement. Hence, four models for anxiety were established. Model 1: Age and sex were considered; Model 2: BMI was added to Model 1; Model 3: Smoking, physical exercise (occasions/week), physical exercise (min/week), and insomnia were added to Model 2; Model 4: Social interaction (occasions/week) was added to Model 3. Using individual sports groups and team sports groups as the between-group factors, the odds ratio (OR) of the relief of anxiety and the improvement of sleep quality was calculated. The intervention effects of individual sports groups and team sports groups were compared according to the OR value.

## 3. Results

### 3.1. Participants’ Characteristics

The baseline participants’ characteristics of the study samples included in the study are shown in Table 1. The mean age of the study sample was 21.3 years, the majority were male (58.4%), and most subjects had a BMI above the normal range. Most of the subjects’ anxiety symptoms had occurred for less than 1 year, and most subjects had self-reports of insomnia. Most of the subjects’ anxiety symptoms were mild anxiety symptoms. The relevant statistical results are shown in Table 1.

### 3.2. The Relief of Anxiety and the Improvement of Sleep Quality in College Students after 6 Weeks’ Intervention

It can be seen from Table 2 that the relief rate of anxiety symptoms in the control group was 38.8%; the relief rate of anxiety symptoms in the individual sports group was 59.4%; and the relief rate of anxiety symptoms in the team sports group was 69.7%. Overall, the team sports group had higher odds of anxiety relief. An analysis of sleep quality showed that the improvement rate of sleep quality in the control group was 28.4%; the improvement rate in the individual sports group was 78.1%; and the improvement rate in the team sports group was 78.8%. Overall, there was no significant difference between team sports groups and individual sports groups in the improvement of sleep quality.

### 3.3. Comparison of Anxiety Symptom Relief Effect and Sleep Quality Improvement Effect among College Students

It can be seen from Table 3 that, in the adjusted Model 1 and Model 2, there were significant differences between the control group and the intervention group, indicating that both team sports and individual sports had an impact on anxiety symptoms and sleep quality.

### 3.4. Build and Revise Models

To better compare the intervention effects of the team sports group and the individual sports group, other influencing factors were included in the study, and then four models were established. According to Table 4, we established four models for the relief effect of anxiety symptoms and sleep quality improvement: Model 1: Age and sex were considered; Model 2: BMI was added to Model 1; Model 3: Smoking, physical exercise (occasions/week), physical exercise (min/week), and insomnia were added to Model 2; Model 4: Social interaction (occasions/week) was added to Model 3. No matter which covariate was added, the intervention effect of the team sports group was better than that of the individual sports group, and there was a significant difference between team sports and individual sports. For the improvement effect of sleep quality, no matter which covariate was added, the improvement effect of individual sports groups was not significantly different from that of the team sports groups.

### 3.5. Summary

**Figure 2:**
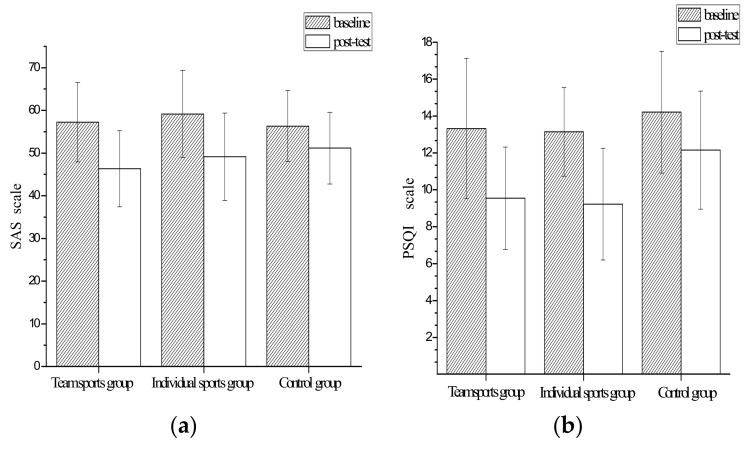
Scores of anxieties and sleep quality in three groups: (**a**) SAS score; (**b**) PSQI score.

According to the analysis, we found that both individual sports and team sports could effectively relieve the anxiety symptoms of college students. However, the team sports group had higher odds of anxiety relief (Figure 2a). In the final model, we found that both individual sports and team sports could effectively relieve the anxiety symptoms of college students, with OR values of 3.18 and 4.99, and with significant difference (*p* = 0.003). As for the improvements to the sleep quality of college students, both individual sports and team sports improved the sleep quality of college students, but the improvement was not too different (Figure 2b). In the final model, we found that both individual sports and team sports effectively improved the sleep quality of college students, with OR values of 7.32 and 7.98, respectively, and there was no significant difference between them (*p* = 0.21). Therefore, according to this study, we determine that team sports have higher odds of anxiety relief, and individual sports and team sports have the same effect on improving sleep quality.

## 4. Discussion

This study examined the effects of individual sports and team sports on anxiety symptoms and sleep quality improvement. The findings of this study were partially consistent with our research hypothesis. On the one hand, this paper has shown that both individual sports and team sports effectively relieved the anxiety symptoms and improved the sleep quality of college students with different effects, and that the team sports group was better than the individual sports group in relieving the anxiety of college students. On the other hand, there was no significant difference between individual sports and team sports in improving the sleep quality of college students, as the improvement effect of sleep quality did not differ according to the type of exercise. A possible reason for this is that sleep quality is only related to physical exercise, rather than the type of it. From the study, we found that team sports such as basketball and football could be appropriately introduced to alleviate the anxiety of college students. In terms of improving sleep quality, our research results found that both team sports and individual sports improved college students’ sleep quality. If college students have both anxiety and sleep quality problems, team sports can be used to intervene in college students for symptom amelioration. In short, this paper found that, although the team sports and individual sports intervention effects were inconsistent, team sports are more suitable for college students to improve anxiety and sleep quality.

The effect of physical exercise on relieving anxiety symptoms in people has been demonstrated [35]. Murphy’s study pointed out that participating in out-of-school physical exercise could effectively relieve the anxiety level of high school students, compared with individuals who did not participate in out-of-school physical exercise [36]. Therefore, whether people participate in spontaneous or non-spontaneous physical exercise, both forms of physical exercise can effectively relieve anxiety levels. Johnston used team sports and aerobic dance classes to compare the intervention effects of anxiety and the depression symptoms of college students. The study found that team sports were better than aerobic dance classes in relieving the depression symptoms of college students, but there was little difference between the team sports and aerobic dance classes for the anxiety symptoms of college students [37]. This is in contrast to the present study, where our research points out that team sports can be more effective in relieving anxiety symptoms in college students. The reason why we have different research results from Johnston can be explained as follows: the exercise intensity and exercise programs adopted were different. Moderate/high-intensity individual sports were used in our study, while aerobic dance is a moderate-intensity exercise. Moreover, the actions we set were relatively simple and could be exercised independently: college students could exercise according to their requirements without communication. However, aerobic dance classes may require learning movements, and there may have been some learning exchanges between college students, which may have caused our research results to be inconsistent with Johnston’s research results. In our study, team sports also had various forms of communication during exercise, and the team sports group also had more in-depth communication in the last 10 min of each session. Communication among college students may be the reason for the differences in anxiety symptoms between team sports and individual sports. There are also differences in anxiety among professional athletes in different sports. Anxiety symptoms of athletes are mainly derived from the pressure of competition. Athletes need to perform effectively at their sport, but sometimes they lack confidence, leading to anxiety symptoms. As Pluhar points out, individual sports athletes are nearly twice as likely to experience anxiety symptoms as team sports athletes [27]. The possible reason is that individual sports are mainly goal-oriented based on completing tasks, while team sports are not only goal-oriented to complete tasks but also goal-oriented based on interests. Another reason may be that team sports athletes require more emotional communication and encouragement among teammates during training, so anxiety symptoms are lower than for individual sports athletes. Our study of college students’ sleep quality found no significant differences between team sports groups and individual sports groups, possibly because improving sleep quality did not require social interaction. One study indicated that 12 weeks of high-intensity interval training had a certain improvement in sleep quality in adults [38]. Lazaridou pointed out that yoga training effectively improved the sleep quality of their subjects [39], and the study of Gumus Sekerci proved that walking exercise can also improve the sleep quality of the elderly [40]. Therefore, we can see that low-intensity physical exercise can also improve individual sleep quality. The mechanism of the effect of low-intensity physical exercise on sleep quality can be attributed to the fact that low-intensity physical exercise can also bring on fatigue, thus improving sleep quality [41]. Rosa applied ball games and judo to intervene for children and adolescents. Studies have found that both ball games and judo can effectively improve the sleep quality of children and adolescents, but ball games have a better effect on improving sleep quality in children and adolescents. The explanation is that ball games may enhance children’s interest and excitement, and result in more fatigue at the end of the exercise [42]. Our study on sleep quality is consistent with Rosa’s findings. As to why team sports and individual sports have the same effect on sleep quality, a possible reason is that fatigue is a key factor in improving sleep quality and participating in physical exercise can make people feel physically fatigued, thus improving the sleep quality of the subjects. The additional social communication of physical exercise does not affect physical fatigue, so individual sports and team sports have no significant difference in sleep quality.

Of course, our study also has shortcomings, the first of which is that the results obtained were all self-assessment scales, and thus were subject to the subjectivity of college students when filling out the questionnaires. The second point is that it is difficult to grasp the intensity of exercise intensity for team sports. Since the team sports group measures were competitions, they involved moderate/high-intensity exercise most of the time, but the training volume of the team sports group was more difficult to monitor than the training volume of the individual sports group. Thirdly, we did not apply an actigraphy device in this study. Wearing a heart rate monitor or some other actigraphy device (such as a watch) could help to grasp the intensity of exercise, in addition to helping to monitor and analyze daily sleep patterns. Additionally, we also did not group anxious college students based on their period of having anxiety. In future studies, we will study the proportion of anxiety improvement after physical exercise in college students who have suffered from anxiety for one year, changed anxiety symptoms for two years, or more than two years to determine the specific intervention effect of physical exercise.

## 5. Conclusions

Six weeks of individual sports and team sports can effectively relieve college students’ anxiety and improve their sleep quality. However, the intervention effects of team sports and individual sports on the anxiety and sleep quality of college students were different. We found that team sports were better than individual sports in relieving the anxiety of college students, and there was a significant difference. For sleep quality, team sports and individual sports had the same improvement effect, and there was no significant difference between the team sports group and the individual sports group. Through this study, we believe that team sports should be used as much as possible to relieve the anxiety symptoms of college students.

## Figures and Tables

**Table 1:** The basic datasheet for the three groups.

Characteristic	Team Sports Group		Individual Sports Group		Control Group		*p*-Value
	Mean [SD] or *n* (%)	*n*	Mean [SD] or *n* (%)	*n*	Mean [SD] or *n* (%)	*n*	
Age (years)	21.42 [2.4]	66	22.31 [3.1]	64	21.90 [2.1]	67	0.87
Male/Female	35/31 (53.0/47.0)	66	39/25 (60.9/39.1)	64	41/26(61.2/38.8)	67	0.57
BMI (kg/m^2^)	27.43 [5.5]	66	27.82 [4.9]	64	27.50 [5.0]	67	0.79
Body fat rate	29.43 [5.2]	66	30.22 [6.3]	64	28.91 [4.9]	67	0.66
Smoking	9 (13.6)	66	6 (9.4)	64	11 (16.4)	67	0.43
Year with anxiety (<1 year)	57 (86.4)	66	59 (7.8)	64	55 (82.1)	67	0.42
1–2 years	8 (12.1)	66	5 (33.3)	64	12 (17.9)	67	0.32
>2 years	1 (1.5)	66	0 (0.0)	64	0(0.0)	67	0.43
insomnia	40 (60.6)	66	42 (65.6)	64	45 (67.2)	67	0.49
Physical exercise (occasions/week)	0.71 [1.2]	66	0.80 [0.9]	64	0.52 [1.1]	67	0.57
Physical exercise (min/week)	32.43 [17.3]	66	43.18 [13.5]	64	40.42 [18.3]	67	0.60
Social interaction (occasions/week)	0.31 [0.53]	66	0.46 [0.67]	64	0.25 [0.72]	67	0.49
Rating scale scores PSQI	14.32 [3.8]	66	13.14 [2.4]	64	14.21 [3.3]	67	0.32
Rating scale scores SAS	57.23 [9.3]	66	59.14 [10.2]	64	56.32 [8.3]	67	0.63

Abbreviations: BMI—Body Mass Index; PSQI—Pittsburgh Sleep Quality Index; SAS—Self-Rating Anxiety Scale.

**Table 2:** The relief of anxiety and the improvement of sleep quality in college students after 6 weeks of intervention.

	Team Sports Group	Individual Sports Group	Control Group
SAS scores			
All, *n*	66	64	67
Improvement, *n* (%) ^a^	46 (69.7)	38 (59.4)	26 (38.8)
No improvement, *n* (%)	20 (30.3)	26 (40.6)	41 (61.2)
PSQI scores			
All, *n*	66	64	67
Improvement, *n* (%) ^b^	52 (78.8)	50 (78.1)	19 (28.4)
No improvement, *n* (%)	14 (21.2)	14 (21.9)	48 (71.6)

Abbreviations: ^a^—relief in anxiety symptoms defined as the decrease in SAS scores of >6 points; ^b^—improvement in sleep quality defined as the decrease in PSQI scores of >2 points.

**Table 3:** Effect of inter-group self-evaluation on depressive symptoms and anxiety symptoms.

Change in SAS Scores		Effect Size (95% CI)	*p*-Value
Model 1 ^a^			
Control vs. Individual sports (*n* = 131)		11.32 (5.32–17.46)	0.006
Control vs. Team sports (*n* = 133)		12.28 (5.78–18.65)	0.002
Model 2 ^b^			
Control vs. Individual sports (*n* = 131)		12.99 (6.03–18.90)	0.001
Control vs. Team sports (*n* = 133)		13.68 (6.27–19.11)	0.001
Change in PSQI scores			
Model 1 ^a^			
Control vs. Individual sports (*n* = 131)		5.13 (1.21–8.79)	0.005
Control vs. Team sports (*n* = 133)		5.35 (1.36–9.21)	0.002
Model 2 ^b^			
Control vs. Individual sports (*n* = 131)		5.86 (2.17–9.78)	0.002
Control vs. Team sports (*n* = 133)		6.32 (2.89–10.32)	0.000

Abbreviations: ^a^—adjusted for sex and age; ^b^—adjusted for sex, age, BMI, body fat rate, smoking, years with anxiety, insomnia, physical exercise (occasions/week and min/week), and social interaction at baseline.

**Table 4:** Established models and revised models.

	Model 1 ^a^		Model 2 ^b^		Model 3 ^c^		Model 4 ^d^	
	OR (95% CI)	*p* (*n*)	OR (95% CI)	*p* (*n*)	OR (95% CI)	*p* (*n*)	OR (95% CI)	*p* (*n*)
SAS scores								
Individual sports	2.64 (2.17–9.78)	0.02 (197)	2.86 (2.32–10.21)	0.01 (197)	3.05 (2.68–10.78)	0.03 (197)	3.18 (2.87–11.21)	0.01 (197)
Team sports	4.05 (3.05–12.03)	0.01 (197)	4.49 (3.67–13.85)	0.005 (197)	4.89 (3.96–14.11)	0.01 (197)	4.99 (4.06–14.87)	0.003 (197)
Compared	1.78 (2.26–9.93)	0.01 (197)	2.04 (2.69–10.80)	0.004 (197)	2.79 (2.97–11.84)	0.013 (197)	2.90 (2.99–12.00)	0.003 (197)
PSQI scores								
Individual sports	5.78 (4.32–17.03)	0.006 (197)	6.32 (4.89–17.87)	0.01 (197)	6.98 (5.02–17.95)	0.01 (197)	7.32 (5.35–18.22)	0.005 (197)
Team sports	5.96 (4.49–18.42)	0.002 (197)	6.78 (5.76–19.13)	0.005 (197)	7.09 (6.16–19.66)	0.004 (197)	7.98 (6.69–19.98)	0.003 (197)
Compared	5.73 (4.29–16.32)	0.41 (197)	5.94 (4.94–17.12)	0.30 (197)	6.05 (5.01–17.79)	0.33 (197)	6.96 (6.19–18.02)	0.21 (197)

Abbreviations: ^a^—age and sex were considered; ^b^—age, sex and BMI were considered; ^c^—age, sex, BMI, smoking, physical exercise (occasions/week and min/week), and insomnia were considered; ^d^—age, sex, BMI, smoking, physical exercise (occasions/week and min/week), insomnia, and social interaction (occasions/week) were considered.

## Data Availability

The data presented in this study are available on request from the corresponding author. The data are not publicly available due to privacy reasons.
